# Penology

**Published:** 1896-03-07

**Authors:** 


### Modern Sociology.

### PENOLOGY.

Punishment is a sad necessity. We are by no means all good, and in every country law-breakers have, by some means or other, to be compelled to conform to the will of the ruling majority. The rough-and-ready method which we inherit from the ancients is the simple plan of punishing the offender, hoping that by observation of his evil fate others will be deterred from walking in his footsteps. Even from early times, linked with ideas of punishment, there have been vague notions of prevention; but it cannot be doubted that of old, and far too much even now, the deep down, bottom principle in the action of the law was, and is, punishment and revenge.

Since the days of Howard, however, the treatment of criminals has come to be looked on with somewhat different eyes. The restoration of the criminal to decent life, and the prevention of the very origins of crime, have come to be regarded as even more important than the mere punishment of the individual, and we are introduced to a new branch of knowledge—penology or the science of punishment, closely linked in practice with that of the prevention of crime. A most interesting book has been recently written by Mr. William Tallack, secretary of the Howard Association, on "Penological and Preventive Principles,"* a perusal of which will be sufficient to convince anyone of the fact that, however great may have been the improvement during recent years in the methods adopted for the treatment of criminals, much yet remains to be done before it can be considered completely satisfactory.

The verdict of a jury is proverbially an uncertain thing; but even more uncertain seems the punishment meted out to those that are found guilty. It will be generally agreed that what is wanted is (1) certainty that punishment will follow crime; and (2), for the control of habitual offenders, who, after all, are the most difficult to deal with, certainty that repeated crime will be followed by progressively increased sentences, even although such sentences might only take the form of liability to, rather than actual imprisonment, combined of course with supervision. Yet it has been shown by official statistics that the graver sentences upon English criminals have often been to a large extent a matter of chance, that a sort of stereotyped conventional fashion has prevailed with the judges in this matter, and that they have glided into a customary groove, marked by certain favourite periods especially five, seven, ten, and fifteen years, without any attempt to proportion the punishment to the crime with any degree of nicety. In proof of this Sir E. F Du Cane has quoted official statistics showing that in a recent period the average number of prisoners under sentences of five years was 2,043, but of those for six years only 43. There was an average of 4,703 for seven years, and of only 366 for eight years; 1,893 for ten years, and none at all for eleven years. Hence great cruelty and injustice may often have been perpetrated upon unfortunate wretches through this mere habit on the part of the judges. But even when the sentence has been given there is but small certainty as to its severity being really what has been intended, so much depends on the individuals by whom it is carried out, an imprisonment of one year under one set of custodians being as really penal as double or treble the same period under the care of another body of officers.

One of the greatest causes of diversity of punishment for the same offence arises from the fact that forms of imprisonment which to the hardened offender may be matters of indifference and entirely devoid of deterrent influence are, to more sensitive natures, punishments of extreme severity. This is due to the custom of allowing prisoners to congregate together during their confinement, a system which makes prisons pleasant places of resort to habitual criminals, while it turns them into places of torment to what one may call accidental offenders. This system has for a long time largely given way in England to the separate system for short-time prisoners, and for the early portion of long sentences in all cases, but it is still prevalent, and even general, in most European Colonial, American, and other jails.

But even comparatively recently it had not quite died out in England, for so lately as 1887 a committee appointed by the Home Secretary laid bare a shocking series of abuses in English jails, and especially in the places where untried prisoners were detained, it being reported that in some courts "the worst evils of that promiscuous association, against which it has been a primary object of modern prison discipline to guard, must be encountered for hours and even days together by children, women, and men, who may be, and some of whom are, innocent." In other places, the persons awaiting trial were kept separate "but by means which appear to be capable of amounting to positive torture," that is to say, by locking them up in narrow cells, or rather cupboards, less than a yard square! Many of these cells had no seats for inmates, others only seats of stone or iron. The most disgusting offences against decency are detailed, and it is said "in such places and under such conditions, for hours or days, children and young women, many of them virtuous and absolutely innocent, have been crowded together with thieves, prostitutes, and all manner of vile characters." In some of these places even the elementary decency of separation of the sexes was not provided. It may be said that this was some time ago, but as lately as 1889, in a prison of good repute, that of Manchester, a man, undergoing a short term of imprisonment for being drunk and disorderly, was cruelly "done to death" while shut up during the night with five other prisoners and one officer, and never yet has the perpetrator of that deed been discovered. The large prisons of England have, however, been greatly improved, and every effort is made to render them as human and as reformatory as possible. Foreign prison systems are extremely various, as we have shown in a former article, ranging from the grossest cruelty to a leniency which is absolutely absurd.

* "Penological and Preventive Principles." London: Wertheimer, Lea, and Co. Price 8s.

